# Transcriptome responses to *Ralstonia solanacearum* infection in the roots of the wild potato *Solanum commersonii*

**DOI:** 10.1186/s12864-015-1460-1

**Published:** 2015-03-26

**Authors:** A Paola Zuluaga, Montserrat Solé, Haibin Lu, Elsa Góngora-Castillo, Brieanne Vaillancourt, Nuria Coll, C Robin Buell, Marc Valls

**Affiliations:** Genetics Department, Universitat de Barcelona and Centre for Research in Agricultural Genomics (CSIC-IRTA-UAB-UB) Edifici CRAG, Campus UAB, Bellaterra, 08193 Catalonia, Spain; Department of Plant Biology, Michigan State University, East Lansing, MI 48824 USA

**Keywords:** *Solanum commersonii*, Bacterial wilt, Brown rot, *Ralstonia solanacearum*, RNA sequencing, Transcriptome, Disease resistance

## Abstract

**Background:**

*Solanum commersonii* is a wild potato species that exhibits high tolerance to both biotic and abiotic stresses and has been used as a source of genes for introgression into cultivated potato. Among the interesting features of *S. commersonii* is resistance to the bacterial wilt caused by *Ralstonia solanacearum,* one of the most devastating bacterial diseases of crops.

**Results:**

In this study, we used deep sequencing of *S. commersonii* RNA (RNA-seq) to analyze the below-ground plant transcriptional responses to *R. solanacearum*. While a majority of *S. commersonii* RNA-seq reads could be aligned to the *Solanum tuberosum* Group Phureja DM reference genome sequence, we identified 2,978 *S. commersonii* novel transcripts through assembly of unaligned *S. commersonii* RNA-seq reads. We also used RNA-seq to study gene expression in pathogen-challenged roots of *S. commersonii* accessions resistant (F118) and susceptible (F97) to the pathogen. Expression profiles obtained from read mapping to the *S. tuberosum* reference genome and the *S. commersonii* novel transcripts revealed a differential response to the pathogen in the two accessions, with 221 (F118) and 644 (F97) differentially expressed genes including *S. commersonii* novel transcripts in the resistant and susceptible genotypes. Interestingly, 22.6% of the F118 and 12.8% of the F97 differentially expressed genes had been previously identified as responsive to biotic stresses and half of those up-regulated in both accessions had been involved in plant pathogen responses. Finally, we compared two different methods to eliminate ribosomal RNA from the plant RNA samples in order to allow dual mapping of RNAseq reads to the host and pathogen genomes and provide insights on the advantages and limitations of each technique.

**Conclusions:**

Our work catalogues the *S. commersonii* transcriptome and strengthens the notion that this species encodes specific genes that are differentially expressed to respond to bacterial wilt. In addition, a high proportion of *S. commersonii*-specific transcripts were altered by *R. solanacearum* only in F118 accession, while phythormone-related genes were highly induced in F97, suggesting a markedly different response to the pathogen in the two plant accessions studied.

**Electronic supplementary material:**

The online version of this article (doi:10.1186/s12864-015-1460-1) contains supplementary material, which is available to authorized users.

## Background

*Ralstonia solanacearum* is the causal agent of the destructive bacterial wilt disease in tropical and subtropical crops, including tomato, tobacco, banana, peanut and eggplant [1]. *R. solanacearum* is one of the most aggressive bacterial pathogens infecting potato (*Solanum tuberosum* L.). The disease in potato is also called brown rot and is endemic in the Andean region, where potato is a staple food, causing an important impact on food production, public health and the economy of the region [2,3]. The pathogen is transmitted by soil, water or infected material; it invades the plant through wound sites in the roots and rapidly colonizes the xylem vessels, where it produces large amounts of exopolysaccharides that block water flow causing wilting and eventually plant death [4].

Durable resistance against *R. solanacearum* in cultivated potato or in any of the commercial varieties of other hosts is scarce, rendering the control of bacterial wilt challenging [5]. Loci or genes for quantitative resistance to bacterial wilt have been recently identified in tobacco [6], tomato [7,8], eggplant [9] and in the model species *Medicago truncatula* [10]. However, there is limited knowledge on the molecular basis of these resistances. The best characterized resistance response to *R. solanacearum* is mediated by RRS1-R, a single *Arabidopsis thaliana* gene encoding a TIR-NBS-LRR protein which is able to recognize the bacterial effector PopP2 and provide recessive resistance [11-13].

Potato breeding programs have used wild species related to *S. tuberosum* as a source of resistance against bacterial wilt [14-16]. Initially, *S. phureja* was used to successfully introgress resistance in potato against *R. solanacearum* [16,17]. Nonetheless, this germplasm shows resistance at high altitudes yet becomes susceptible when grown at warmer temperatures in the lowlands [17,18], suggesting the existence of latent infections (i.e., infected plants that remain asymptomatic [19]). Despite this drawback, the use of resistant varieties is an important approach to control the disease. *S. commersonii* Dun [20], native to Uruguay, Argentina and Brazil, has been used as a valuable source of resistance to several diseases including bacterial wilt [14,15,21-25]. This wild relative of potato is diploid, and has shown segregation of resistance against bacterial wilt [22]. Gonzalez et al. [22] obtained a *S. commersonii* population (accessions F1 to F121) that segregated for *R. solanacearum* resistance, suggesting polygenic control for this trait.

Applying transcriptomics to study host-pathogen interactions has provided unparalleled insight into the mechanisms underlying disease development, basal defense, and gene-for-gene resistance. For instance, seminal work on genome-wide expression studies revealed important overlaps in plant gene expression at the early stages of incompatible interactions and the late stages of compatible interactions [26]. In potato, microarray studies on resistant and susceptible cultivars have shown that infection with *Rhizoctonia solani* [27], *Phytophthora infestans* [28] and potato virus Y [29] induce both general and cultivar-specific defence genes and systemic resistance. In a recent report, transcriptomic comparison of potato varieties resistant or susceptible to the late blight pathogen *P. infestans* enabled the identification of candidate genes for quantitative resistance to this disease [30]. Transcriptional responses in leaves associated with bacterial wilt disease development were studied in-depth for the model plant *A. thaliana* [31]. This study showed little impact of the pathogen at the early infection stages and up-regulation of ABA, senescence and basal resistance-associated genes during wilting. Similarly, the transcriptome of two tomato cultivars with contrasting resistance against *R. solanacearum* identified pathogenesis-related, hormone signaling and lignin biosynthesis genes induced in stems of the resistant cultivar LS-89 while no change in gene expression was detected for the susceptible cultivar Ponderosa [32]. Regarding potato responses to bacterial wilt, a cDNA-AFLP approach was used to isolate specific transcripts expressed in the aerial parts during resistant and susceptible interactions, revealing metabolites exclusively produced in the resistant genotypes [33]. More recently, using *S. tuberosum* cDNA microarrays, the transcriptome of the highly-resistant *S. commersonii* accession F100 was determined in stem tissue 6 to 120 hours after challenge with *R. solanacearum* [15]. These results suggest a role of both salicylic acid (SA) and ethylene (ET) in the early defense responses [15], but the array technology used did not provide information on any *S. commersonii* lineage-specific genes that may contribute to resistance against *R. solanacearum*. As knowledge in plant defense at the root-microbe interface is scarce, we have characterized in this study *R. solanacearum*-potato interactions by examining the below-ground transcriptome of two additional *S. commersonii* accessions from the aforementioned cross that show susceptibility (F97) and resistance (F118) to bacterial wilt. We employed sequencing of RNAs (RNA-seq) to compare the root transcriptome of pre-symptomatic plants colonized by *R. solanacearum* to that of non-inoculated plants. RNA-seq has three important advantages compared to microarray hybridization: it detects all existing transcripts irrespective of gene annotation, it is a direct quantitative measure of gene expression and it is more sensitive to lowly-expressed transcripts [34]. We describe for the first time the *S. commersonii* transcriptome response to *R. solanacearum* including the identification of candidate genes and novel *S. commersonii* transcripts that may have a role in resistance in this wild potato species to bacterial wilt. Additionally, we compare two different methods to eliminate ribosomal RNA to obtain plant-derived mRNAs for RNA seq.

## Results and discussion

### Generating a *solanum commersonii* reference sequence from RNA-seq reads

**Figure 1:**
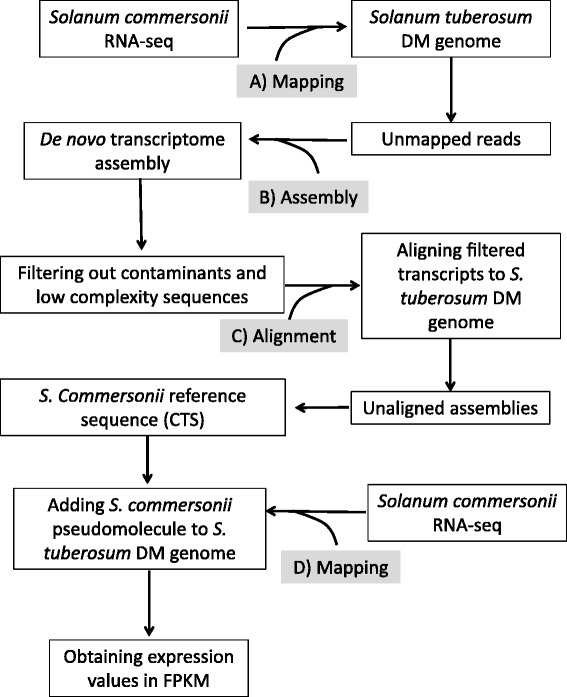
**Workflow of analysis of root RNA samples from the** ***S. commersonii*** **accessions and generation of the** ***S. commersonii*** **reference sequence.** **A)** RNA-seq reads were mapped using TopHat [37] to *Solanum tuberosum* DM genome and unmapped reads were retained. **B)** Unmapped reads were filtered for low quality and artifacts using Cutadapt and FASTX toolkit [71,72]. The cleaned reads were used to carry out a *de novo* transcriptome assembly using Oases [38]. The assembled transcripts were filtered out for low complexity sequences and possible contamination by searching sequences against the Uniref100 database. **C)** High quality transcripts assemblies were aligned to *S. tuberosum* DM genome using Gmap [42] and unaligned transcripts were kept for further analyses. In order to eliminate redundancy only the longest isoform from each Oases locus was used as the representative transcript. Representative transcripts were used to create a reference sequence that was added as an additional chromosome to the *S. tuberosum* DM genome. **D)** *Solanum commersonii* RNA-seq was mapped to the *S. tuberosum* DM genome and *S. commersonii* lineage specific concatenated transcript sequences using TopHat. After mapping, expression values were obtained using Cufflinks [43].

We chose to analyze *S. commersonii* transcriptional responses to *R. solanacearum* in the root tissues, where the infection starts. To this end, we chose to inoculate plants with the highly-aggressive *R. solanacearum* UY031 strain, which was originally isolated from potato [4]. Root infection is a stochastic process that results in high colonization variability in plants soil-inoculated with *R. solanacearum* [19]. In order to standardize infections, a previously developed light-emitting derivative of the UY031 strain [35] was used for plant inoculation and root sections from asymptomatic plants that contained similar bacterial loads were selected after measuring light emission with a luminometer. RNA was extracted from inoculated and non-inoculated *S. commersonii* roots or from a mixture of tissues containing flower, root, stolon, shoot and leaves, and subjected to Illumina sequencing after mRNA enrichment by polyA+ selection or ribosomal RNA depletion (see below). The RNA-seq reads derived from polyA+ selection were used to construct a *S. commersonii* transcriptome assembly that was maximized to identify novel transcripts derived from the two plant genotypes analyzed (F118 and F97). To detect these novel transcripts, RNA-seq reads were first mapped to the *S. tuberosum* Group Phureja DM genome [36] using Tophat [37]. Aligned reads were discarded and un-aligned reads were retained (Figure 1, Table 1) and filtered for quality, resulting in 185,410,176 high quality illumina sequences that were used in a *de novo* transcriptome assembly using the transcriptome assembler Oases [38]. A total of 159,755,431 (86.16%) reads were assembled into 165,668 transcripts including isoforms (Table 2). Possible contaminants in the transcript assemblies were identified and removed by searches against the UniRef100 database [39] using BLASTX [40]. Low complexity sequences (4,896) were removed using a custom perl script [41] resulting in 160,146 high-quality assembled transcripts. Statistics including N50 contig and average transcript length were calculated (Table 2); an N50 contig size larger than 1kb and an average transcript length of 898 nucleotides indicated a robust assembly of the *S. commersonii* transcripts.

**Table 1:** ***S. commersonii*** **RNA-seq library description and number of unmapped reads** to ***S. tuberosum*** **Group Phureja DM genome**

**Genotype**	**Treatment**	**ID**	**Total reads**	**Mapped reads to** ***S. tuberosum*** **Group Phureja DM genome (%)**	**Total mapped reads to** ***S. tuberosum*** **Group Phureja DM genome and** ***S. comersonii*** **pseudomolecule (%)**	**Total unmapped reads to** ***S. tuberosum*** **Group Phureja DM genome and** ***S. comersonii*** **pseudomolecule (%)**
Resistant	Inoculated*	F118-inoc	104.964.466	80,915,976 (77.1%)	93,103,245 (88.7%)	11,861,221 (11.3%)
Resistant	Mock-inoculated*	F118-control	136.121.036	103,742,612 (76.2%)	120,857,786 (88.8%)	15,263,250 (11.2%)
Susceptible	Mock-inoculated*	F97-control	76.995.583	60,065,956 (78.0%)	68,045,457 (88.3%)	8,950,126 (11.6%)
Susceptible	Inoculated*	F97-inoc	150.998.983	117,725,715 (78.0%)	133,722,765 (88.6%)	17,276,218 (11.4%)
Susceptible	Mock-inoculated*	CA+CB:F97-control	115.364.615	89,248,600 (77.4%)	102,851,301 (89.2%)	12,513,314 (10.9%)
Susceptible	Inoculated*	IA+IB:F97-inoc	117.130.841	91,346,770 (78.0%)	104,504,388 (89.2%)	12,626,453 (10.8%)
Susceptible	Pool of RNA **	F97 pool of RNA	206.220.325	167,556,845 (81.2%)	170,530,084 (82.7%)	35,690,241 (17.3%)
Susceptible	Pool of RNA **	F97 pool of RNA	206.220.325	161,702,303 (78.4%)	164,543,511 (79.8%)	41,676,814 (29.2%)

*Total RNA was pooled from root tissue.

**Total RNA was pooled from flower, stolon, stem and infected or non-infected root.

**Table 2:** **Statistics of the** ***S. commersonii*** **de novo transcriptome assembly**

Reads for assembling	185.410.176
Assembled reads	159.755.431
% of assembled reads	86,2
Number of transcripts	160.146
Maximum length (bp)	9.174
Minimum length (bp)	250
Average size of contigs (bp)	898
N50 contig (bp)	1.242

A second step in the pipeline involved the identification of orthologues of *S. tuberosum* Group Phureja with the *S. commersonii* transcript assemblies. The *S. commersonii* transcript assemblies were aligned to the *S. tuberosum* DM genome [36] using Gmap [42]; of these, 148,387 transcripts (92.7%) aligned to the DM genome sequence using 80% identity and 80% coverage as thresholds and were discarded as these are close orthologs with gene in the annotated potato reference genome. The un-aligned transcripts (~8%) which represent putative novel transcripts in *S. commersonii* were used for downstream analysis. As the Oases algorithm generates isoforms of a locus that represents true alternative isoforms, alleles and paralogues, downstream analyses utilized the “representative” transcript that is the longest isoform of loci with more than one isoform, yielding a total of 9,766 representative transcripts [41]. Manual review of the un-aligned transcripts revealed sequences with similarity to proteins from oomycetes, fungi, algae and metagenome projects, suggesting that our initial filtering failed to identify all potential contaminants in the *de novo* assembly. Contaminating RNAs may originate from endophytes living in the rhizosphere or present in the unsterilized soil in which the plants had been grown. Thus, we implemented two additional filters to identify only high confidence novel *S. commersonii* transcripts for use in downstream expression profiling experiments. First, using the BLAST alignments to the UniRef100 database, the stringency for annotating a transcript as a potential contaminant was lowered and any transcript with a top BLAST match to a non-Viridiplantae sequence (<1e-10) was removed. For transcripts lacking similarity to the UniRef100 database, we searched the transcripts against the annotated *S. tuberosum* Group Phureja proteome [36] and removed any transcript that lacked > 50% identity, > 50% coverage with an E-value of <1e-5. A total of 6,788 sequences out of the 9,766 representative transcripts were removed yielding 2,978 representative transcripts that were stitched together by inserting Ns between the representative sequences to generate a set of concatenated transcript sequences (CTS).

**Figure 2:**
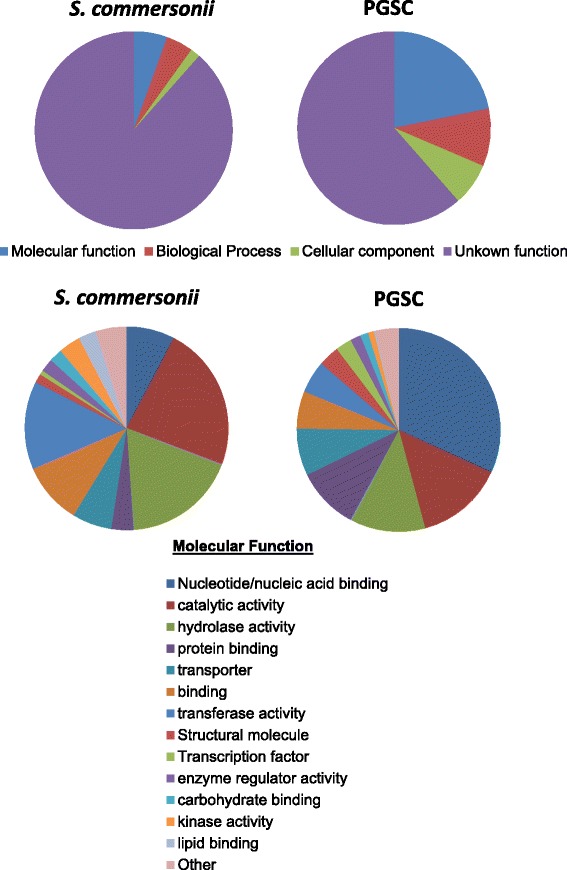
**Gene Ontology category distribution of novel** ***S. commersonii*** **transcripts and predicted genes in the reference** ***S. tuberosum*** **DM genome.** Pie charts on the top show gene distribution according to major Gene Ontology Categories. The bottom charts detail sub-categories of molecular function. Nucleotide/nucleic acid binding includes the categories: Nucleotide binding, DNA binding, Nucleic acid binding and RNA binding. All categories below 2% of representation in both gene sets (signal transducer activity, molecular function, receptor activity, translation factor activity, nuclease activity, chromatin binding, motor activity, receptor binding) were grouped in “Other”. PSGC: Potato Genome Sequence Consortium.

*S. commersonii* is a valuable source for potato breeding programs due to its genetic variability and high resistance to bacterial or fungal pathogens as well as abiotic stress including frost tolerance. Thus, we classified the newly described genes from the *S. commersonii*-specific transcript assemblies using Gene Ontology (GO) Slim terms functional categories and compared them to the *S. commersonii* transcripts with orthologues in the *S. tuberosum* DM genome. The categories in the *S. commersonii* lineage-specific transcripts compared to the annotated DM genome (PGSC) are depicted in Figure 2. Molecular function categories of genes with an enzymatic activity (catalytic activity, hydrolase activity, transferase activity) were the most represented (55.2%) in the *S. commersonii-*specific transcripts vs 31.3% in the DM potato genome (Figure 2). Interestingly, analysis of the annotations of the novel *S. commersonii* transcripts revealed that nearly 3% corresponded to biotic/abiotic stress response genes and 1.4% to genes encoding resistance-like proteins. Hence, the *S. commersonii*-specific transcripts, among which, likely lie agronomically valuable genes are described and catalogued for the first time with this work.

### *R. solanacearum* infection preferentially impacts the *S. commersonii-*specific genes and triggers stress responses

Once we had created a set of *S. commersonii*-specific transcripts, we analyzed the root transcriptomes in two accessions of this wild potato species that show contrasting resistance towards bacterial wilt. To this end, RNA-seq reads were mapped to the *S. tuberosum* Group Phureja DM genome [36] and the *S. commersonii* CTS using Tophat [37] and expression abundances in fragments per kilobase exon model (transcript) per million mapped reads (FPKM) were calculated using Cufflinks [43]. Inclusion of the *S. commersonii* CTS resulted in mapping of >88% of the reads (Table 1), clearly improving the efficiency obtained using the DM genome alone as a reference. Interestingly, 33 genes were present in all root samples within the 100 highest expressed (Additional file 1: Table S1), and 18 of these genes were also amongst the 100 highest expressed genes in samples from other non-inoculated pooled potato tissues (Additional file 1: Table S1, shadowed). For functional annotation of these genes we used GO (see Methods). In addition to housekeeping genes, the highly expressed genes present in all tissues included metallothioneins, a catalase, late embryogenesis abundant protein 5 and a glycine-rich RNA binding protein, all involved in stress tolerance. Furthermore, the 15 highly expressed root-specific genes include drought-induced protein SDi and dehydrin DHN10 (Additional file 1: Table S1). The abundance of all of these transcripts in uninfected and unstressed *S. commersonii* plants may reflect the capacity of this wild potato to cope with different environmental stresses.

**Figure 3:**
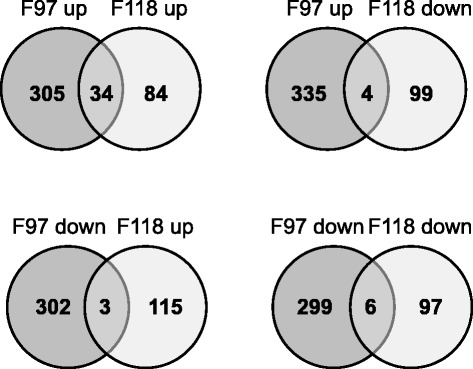
**Genes differentially expressed upon** ***R. solanacearum*** **challenge in the potato genotypes F118 (resistant) and F97 (susceptible).** Venn diagrams showing unique and common up-regulated (up) and down-regulated (down) in inoculated vs non-inoculated control plants.

Differentially expressed genes between treatments were identified using Cuffdiff [43]. Comparison between non-inoculated and pathogen-inoculated roots of the resistant F118 genotype revealed 221 differentially expressed genes (four-fold change and False Discovery Rate FDR < 0.05). Among these genes, a 1,3-beta glucosidase, a threonine dehydratase and an unknown protein were induced the highest, while an ABC transporter, a cysteine protein, a cytochrome P450 and a Hsp90 were highly repressed after pathogen infection (Additional file 1: Table S2 and Figure 3). When inoculated and control roots of the F97 susceptible genotype were compared, 644 differentially expressed genes were detected. An ethylene-responsive transcription factor, Hsp90, heavy metal detoxification, senescence-related and polygalacturonase-encoding genes showed the highest expression levels while genes encoding glycine-rich, non-specific lipid transport and unknown function proteins showed lowest expression after pathogen inoculation (Additional file 1: Table S3 and Figure 3). The two accessions were compared (F97 vs F118) for both non-inoculated and both inoculated roots tissues using the same thresholds as above (Additional file 1: Table S4 and S5). The higher transcriptional response in the susceptible accession and the fact that 1,201 genes showed differential expression between the two inoculated accessions (Additional file 1: Table S5 and Figure 3) indicate that these *S. commersonii* genotypes might have contrasting responses towards *R. solanacearum* in roots. Interestingly, both accessions showed a similar proportion of up- and down-regulated genes after bacterial inoculation (118 up vs 103 down in F118 and 339 up vs 305 down in F97; Figure 3), in contrast with the higher number of down-regulated genes detected in leaves of the highly resistant genotype F100 in a previous microarray study at earlier times of the interaction [15]. Altogether, around 2% of all *S. commersonii* genes were differentially expressed in any of the infected asymptomatic accessions compared to non-inoculated plants. This contrasts with the study by Hu and colleagues [31], where no genes were differentially expressed at early stages of the *A. thaliana-R. solanacearum* interaction. This difference is likely due to the fact that we studied roots, the entry tissue in direct contact with the pathogen, while Hu et al. [31] also infected the roots but performed their transcriptome analyses from leaf tissue. Differences could also be attributable to the nature of the interactions with *R. solanacearum*: quantitative resistance in *S. commersonii* versus the gene-for-gene resistance in *A. thaliana*.

**Figure 4:**
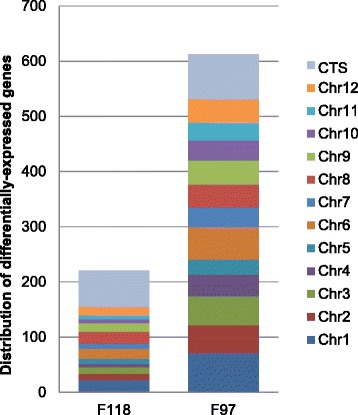
**Abundance of differentially expressed genes by genomic distribution.** Genes differentially regulated upon challenge by *R. solanacearum* are presented according to their genomic location. Chr1 to Chr12 indicate *S. tuberosum* DM chromosomes. CTS indicates genes present in the *S. commersonii* lineage-specific concatenated transcript sequences.

We then analyzed the origin of the differentially expressed genes upon bacterial infection. The chromosomal distribution of these genes in the DM genome or the *S. commersonii* CTS is shown in Figure 4, exceptuating 3 and 32 genes that could not be mapped to a specific chromosome in F118 and F97, respectively. Interestingly, 29.8% of F118 genes up- or down-regulated after inoculation were derived from the *S. commersonii* CTS, in spite of representing only 19.8% of the genes in this species. Over-representation of *S. commersonii* lineage-specific genes in those differentially expressed was not observed in F97. These data support our hypothesis that the *S. commersonii*-specific genes may encode critical genes that function to confer pathogen resistance to F118.

**Figure 5:**
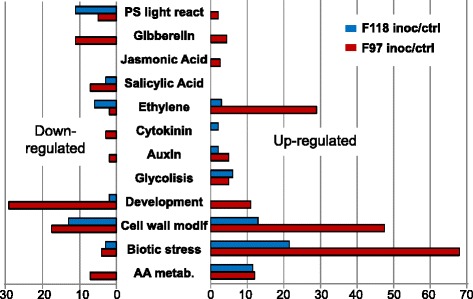
**Comparison of plant responses to bacterial infection in the F118 (resistant) and F97 (susceptible)** ***S. commersonii*** **accessions.** Genes differentially expressed after inoculation with *R. solanacearum* in both accessions were grouped by Gene Ontology functional categories and their abundance represented as bars in the graph. Gene numbers for each category in down-regulated (left panel) and up-regulated (right) genes in the two plant accessions F118 (blue bars) and F97 (red bars) are represented side by side for comparison. Abbreviations are as follows: PS light react: Photosystem light reactions; AA metab: Amino acid metabolism.

**Figure 6:**
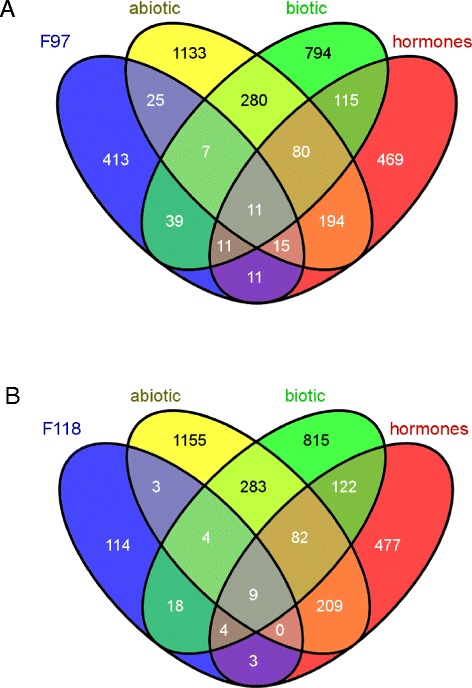
**Comparison of differentially expressed potato genes under various stresses or hormone treatments.** DM-annotated genes differentially expressed following *S. commersonii* root infection with *R. solanacearum* F97 **(A)** and F118 **(B)** accessions were compared to previously-described *S. tuberosum* genes differentially expressed after infection by *Phytophthora infestans* (Biotic), treatment with phytohormone analogues (hormones) or during abiotic stresses (Abiotic) [45]. Common and specific genes to each condition are represented in Venn diagrams.

To obtain a broader picture on the differences in gene expression dynamics in response to pathogen infection in the susceptible (F97) and resistant (F118) genotypes, we categorized the differentially expressed genes (FDR <0.05) according to their gene ontology (GO) annotation (Figure 5). There were two categories (response to biotic stress and cell wall modification) that stood out as up-regulated in both genotypes after inoculation with *R. solanacearum* and one (development) was clearly under-regulated, in line with the accepted notion that induction of plant defense triggers growth inhibition [44]. The category “hormone” was also clearly differentially regulated. We divided this category into genes related to the different phytohormones, which showed that an important number of ethylene-related genes were induced in F97 but not in F118. Similarly, jasmonic acid (JA)-related genes were induced in F97 after pathogen challenge, but not in F118. In contrast, SA-related genes were down-regulated in both accessions (Figure 5). All these differential expression changes were much more apparent in F97, suggesting that this susceptible *S. commersonii* accession is stressed further than the resistant F118 upon pathogen challenge. Since biotic stress genes were clearly up-regulated in both accessions challenged with *R. solanacearum*, we compared differentially expressed genes from our RNA-seq experiments to previous studies of disease resistance in potato. Published transcriptional studies in DM potato defined sets of genes whose expression was specifically triggered by biotic or abiotic stress or by hormone treatment [45]. Comparison of the expression of the DM-annotated genes from our study with those reported in [45], revealed that there is an overlap between *S. commersonii* genes differentially expressed upon *R. solanacearum* inoculation and those of *S. tuberosum* challenged with *P. infestans* or phytohormone analogues (Figure 6). Precisely, 22.6% of the F118 and 12.8% of the F97 genes differentially expressed have been previously identified as responsive to biotic stresses. Also, roughly 10% of the *R. solanacearum*-response genes in both accessions were known as affected by abiotic stresses (salt, mannitol and heat) or hormone treatment (abscisic acid, gibberellin, auxin and cytokinine) [46-51]. In summary, we found substantial overlap of our results with genes involved in pathogen and other stress responses (Figure 6), suggesting that potatoes use a common set of genes to cope with biotic and abiotic stresses. This also supports the emerging hypothesis that common response nodes exist in plants to face alterations in their homeostasis.

### A set of core genes involved in plant defense are up-regulated after pathogen inoculation in the susceptible and resistant *S. commersonii* genotypes

We next analyzed the commonalities and differences in the transcriptome of the susceptible (F97) and resistant (F118) *S. commersonii* accessions upon inoculation with *R. solanacearum* (Figure 3). A total of 34 genes were up-regulated in both accessions (Table 3) and six were down-regulated (Table 4). These genes can be considered indicators of the pre-symptomatic responses of *S. commersonii* to bacterial wilt. Their expression is indicated in Tables 3 and 4 as the logarithm of FPKM in inoculated divided by FPKM in non-inoculated roots (log(inoc/ctr)). Interestingly, 22 of the genes up-regulated in both accessions (65%) had been previously described as involved in plant defense [46-51].

**Table 3:** **Genes up-regulated in both F97 and F118 accessions upon** ***R. solanacearum*** **infection**

**Gene name**	**Annotation**	**F118 log(inoc/ctr)**	**F97 log(inoc/ctr)**
**Pathogen-responsive proteins**			
PGSC0003DMG401010492	Acidic class II 1,3-beta-glucanase	3,03	2,45
PGSC0003DMG400010491	Glucan endo-1,3-beta-D-glucosidase	3,86	3,71
PGSC0003DMG400010490	Acidic class II 1,3-beta-glucanase	2,97	2,39
PGSC0003DMG400029830	Glucan endo-1,3-beta-D-glucosidase	2,70	3,08
PGSC0003DMG400019437	Pathogen-and wound-inducible antifungal protein CBP20	2,74	3,79
PGSC0003DMG400044171	P69E protein	2,61	2,44
PGSC0003DMG400045235	P69E protein	2,72	2,35
PGSC0003DMG401003937	P69B protein	2,74	2,05
PGSC0003DMG400003939	Serine protease	2,36	2,12
PGSC0003DMG400030731	Metallocarboxypeptidase inhibitor	2,87	2,79
**Genes related to auxin metabolism**			
PGSC0003DMG401028570	Phosphoglycerate/bisphosphoglycerate mutase family protein	3,10	2,42
PGSC0003DMG402028570	Phosphoglycerate/bisphosphoglycerate mutase	3,12	2,41
PGSC0003DMG400021406	Phytosulfokine peptide	2,97	2,25
PGSC0003DMG400024978	Indole-3-acetic acid-amido synthetase GH3.3	2,02	2,14
**Genes related to cell death or plant defense**			
PGSC0003DMG400002029	Cytoplasmic small heat shock protein class I	2,98	3,55
PGSC0003DMG400002028	Cytoplasmic small heat shock protein class I	2,98	3,55
PGSC0003DMG400002027	Cytoplasmic small heat shock protein class I	2,98	3,55
PGSC0003DMG400019956	Glutathione s-transferase	2,75	3,26
PGSC0003DMG400010798	Staygreen protein	2,78	2,11
PGSC0003DMG400017713	LRR receptor-like serine/threonine-protein kinase	2,68	2,02
PGSC0003DMG400012992	Receptor protein kinase	3,13	2,73
PGSC0003DMG400031325	Nectarin 5	2,50	2,98
**Unknown function & putative proteins**			
PGSC0003DMG400002772	Conserved gene of unknown function	2,59	4,59
PGSC0003DMG400007526	Conserved gene of unknown function	3,44	2,11
PGSC0003DMG400031326	Conserved gene of unknown function	3.74	2,96
PGSC0003DMG400016493	Gene of unknown function	2,46	3,60
PGSC0003DMG400016351	Conserved gene of unknown function	2,68	2,99
PGSC0003DMG400011404	Gene of unknown function	2,24	3,71
**Genes with uncertain function**			
PGSC0003DMG400026590	Major pollen allergen Ory s 1	4,92	2,34
PGSC0003DMG400027031	Hydrogen peroxide-induced 1	3,06	3,03
PGSC0003DMG400011226	Sodium/potassium/calcium exchanger 6	3,10	3,13
sci_locus_30986_iso_1_len_1653_ver_2	Trypsin and protease inhibitor	2,99	2,89
PGSC0003DMG400012987	Threonine dehydratase biosynthetic, chloroplastic	6,88	4,67
PGSC0003DMG400004690	Metal ion binding protein	4,68	3,75

log(inoc/ctr) stands for logarithm of FPKM in inoculated divided by FPKM in non-inoculated roots.

**Table 4:** **Other differentially expressed genes between accessions**

**Gene name**	**Annotation**	**F118 log(inoc/ctr)**	**F97 log(inoc/ctr)**
**Genes up-regulated in F97 and downregulated in F118 upon** ***R. solanacearum*** **infection**			
sci_locus_40613_iso_1_len_1495_ver_2	3-ketoacyl-CoA thiolase 5, peroxisomal n=2 Arabidopsis thaliana RepID=THIK5_ARATH	-4,18	2,42
sci_locus_57525_iso_1_len_447_ver_2	Lipid binding protein, putative Ricinus communis RepID=B9S7C4_RICCO	-3,09	2,47
PGSC0003DMG400006862	Polygalacturonase	-2,86	5,84
PGSC0003DMG400008255	Conserved gene of unknown function	-2,46	3,75
**Genes down-regulated in both F97 and F118 accessions upon** ***R. solanacearum*** **infection**			
PGSC0003DMG400002519	Zinc finger protein	-2,32	-4,86
PGSC0003DMG400020481	14 kDa proline-rich protein DC2.15	-2,67	-6,35
PGSC0003DMG400023764	Globulin	-2,35	-2,95
PGSC0003DMG400002857	Ribonuclease t2	-2,35	-3,43
PGSC0003DMG401019636	Gene of unknown function	-3,09	-2,80
PGSC0003DMG400046602	Conserved gene of unknown function	-2,92	-6,70
**Genes down-regulated in F97 and up-regulated in F118 upon** ***R. solanacearum*** **infection**			
PGSC0003DMG400004820	Electron transporter	3,31	-2,03
PGSC0003DMG400029879	Mechanosensitive ion channel domain-containing protein	2,34	-3,46
PGSC0003DMG400030913	Tuber-specific and sucrose-responsive element binding factor	2,37	-3,68
sci_locus_16164_iso_1_len_515_ver_2	UPI00016437BC related cluster unknown RepID=UPI00016437BC	2,81	-3,22

log(inoc/ctr) stands for logarithm of FPKM in inoculated divided by FPKM in non-inoculated roots.

Ten pathogen-response (PR) genes were present in the 34 up-regulated in both accessions (Table 3). Four of these genes encoded β-1,3-glucanases belonging to the pathogenesis-related protein 2 (PR2) family. These enzymes are assumed to degrade β-1,3-glucans in the pathogen cell walls to protect the host plant [46,47]. Similarly, the up-regulated gene CBP20 belongs to the PR4 family and has shown both antifungal and β-1,3-glucanase activities [48]. Three P69 family genes encoding subtilisin-like proteinases were also up-regulated in both interactions. P69B was suggested to respond to SA and bacterial effector-triggered defense in tomato [49] and its expression in tomato is restricted to roots [50], the tissue studied in this work. Another serine protease was also induced by *R. solanacearum*, suggesting that these proteases may be involved in host defense against this pathogen*.* Finally, up-regulated genes also included a potato metallocarboxypeptidase inhibitor (PCI), which is induced by JA and ABA [51].

Four genes whose expression was triggered by the pathogen in both accessions are related to the auxin pathway (Table 3). The up-regulated GH3.3 gene encodes an enzyme that conjugates amino acids to indole-3-acetic acid (IAA) and was shown to be induced in *A. thaliana* by pathogen and IAA-amino acid formation [52,53]. This gene, together with GH3.5, GH3.6, and the transcript encoding the growth promotion peptide phytosulfokine (PSK), were described as positively regulated in auxin-mediated adventitious root initiation [54,55]. IAA-Asp, one of GH3.3 products, also caused disease promotion via regulation of pathogen virulence gene expression [52]. Likewise, AtPSK acts as a negative regulator of PAMP-triggered immunity to balance the cost of its activation [56]. Eight PSK members are encoded in the *S. tuberosum* genome, four of which were activated in bacteria-challenged F97 while only one in F118. We also found two up-regulated phosphoglycerate/biphosphoglycerate mutases, which are hypothesized to be involved in glycolysis (http://potatometabolicpathways.webs.com/Metabolic_Pathways_of_Diseased_Potato.pdf). However, in soya bean and *A. thaliana*, this gene is strongly induced by auxin and nematodes and its protein product is detected at the root meristem [57].

Eight other *R. solanacearum*-triggered genes were related to plant defense or cell death (Table 3). For instance, three up-regulated small heat shock proteins were found. These proteins may play a role in defense similarly to the small heat shock protein RSI2, which contributes to tomato resistance by stabilizing resistance protein I-2 [58]. Tobacco small heat shock protein Ntshsp17, also induced by *R. solanacearum*, caused delayed wilting symptoms and was required for effector-triggered immunity [59]. One Glutathione S-transferase (GST) was also induced in challenged *S. commersonii*. GSTs use glutathione peroxidase activity to protect cells from oxidative damage in addition to catalyzing GSH conjugation reactions, binding auxin and phenylopropanoid and transporting anthocyanin into the vacuole [60]. Moreover, it is reported that *NbGSTU1*-silenced plants showed enhanced susceptibility to the pathogen *Colletotrichum orbiculare* [61]. Finally, an induced gene encoded a stay-green protein, which is involved in metabolism of chlorophyll during senescence development and is required for AvrRpm1-triggered HR in *A. thaliana* [62]. Two receptor proteins of the FLS2 and EFR family were also present. These receptors are known to be an important component of plant immunity [63]. Finally, Nectarin 5, which has been proposed to function in producing high levels of hydrogen peroxide and protect from microbial infection, was also induced [64].

The above-mentioned genes likely control the core responses of *S. commersonii* towards the pathogen and may explain the higher tolerance to disease of this wild potato with respect to the cultivated *S. tuberosum*. An indication of this is that three of the above mentioned genes, encoding metallocarboxypeptidase inhibitor, indol-3-acetic acid-amido synthetase (GH3.3) and a leucine-rich repeat (LRR) receptor-like protein kinase, were also found up-regulated in the highly-resistant *S. commersonii* genotype F100 at a very early time of six hours after inoculation with *R. solanacearum* [15]. This suggests the importance of these core genes in *R. solanacearum*-plant interactions at early and later stages of the infection.

Upon pathogen challenge, four genes were up-regulated in the susceptible F97 accession and down-regulated in F118 (Figure 3). We analyzed these genes, since they might correspond to bacterial wilt susceptibility genes and therefore could be good candidates to engineer potato varieties resistant to this disease. Two of these genes (3-ketoacyl-CoA thiolase 5 and lipid binding protein) are *S. commersonii*-specific -not present in the *S. tuberosum* Group Phureja DM genome- and belong to the GO category fatty acid metabolism/beta-oxidation, involved in the metabolism of the phytohormone JA (Table 4). 3-ketoacyl-CoA thiolase 5 (KAT5) is a key enzyme in JA biosynthesis and its deletion in *A. thaliana* impaired both JA-mediated resistance to insect and pollen viability [65]. Hormones are known to play a critical role in the outcome of plant-pathogen interactions. Higher levels of JA in the susceptible accession compared to the resistant one might explain the contrasting response against *R. solanacearum*, as JA is known to antagonize SA signaling, which is necessary for systemic resistance [66]. These JA and SA hormone alterations could be specific to potato, as previous studies in *A. thaliana* showed that *R. solanacearum* is able to successfully suppress the SA defense pathway [12,31,67]. The differences in the hormone responses of the two *S. commersonii* accessions could be due to different genetic backgrounds that cause specific plant responses or to pathogen interactions specific to one accession. In this sense, it is worth mentioning that the SA pathway appeared activated upon infection in a microarray gene expression study using the *S. commersonii* resistant accession F100 [15]. Finally, the presence of a polygalacturonase within the differentially expressed genes in the two accessions could be speculated to be an outcome of bacterial virulence. Bacterial and fungal pathogens secrete such enzymes to promote infection and the plant hosts even generate polygalacturonase-inhibiting proteins in response [68]. It could be that *R. solanacearum* hijacks the host enzyme to escape from plant polygalacturonase inhibitors.

An analysis of the genes down-regulated in both accessions or whose transcripts increased in F118 and decreased in F97 upon inoculation (Figure 3) did not reveal genes known to be related to disease responses, but we believe that these sets of genes (Table 4) are also a useful source for future research on plant-pathogen interactions.

### Ribosomal RNA depletion as a method to assess differentially expressed genes in bacterial infected plant tissues

**Figure 7:**
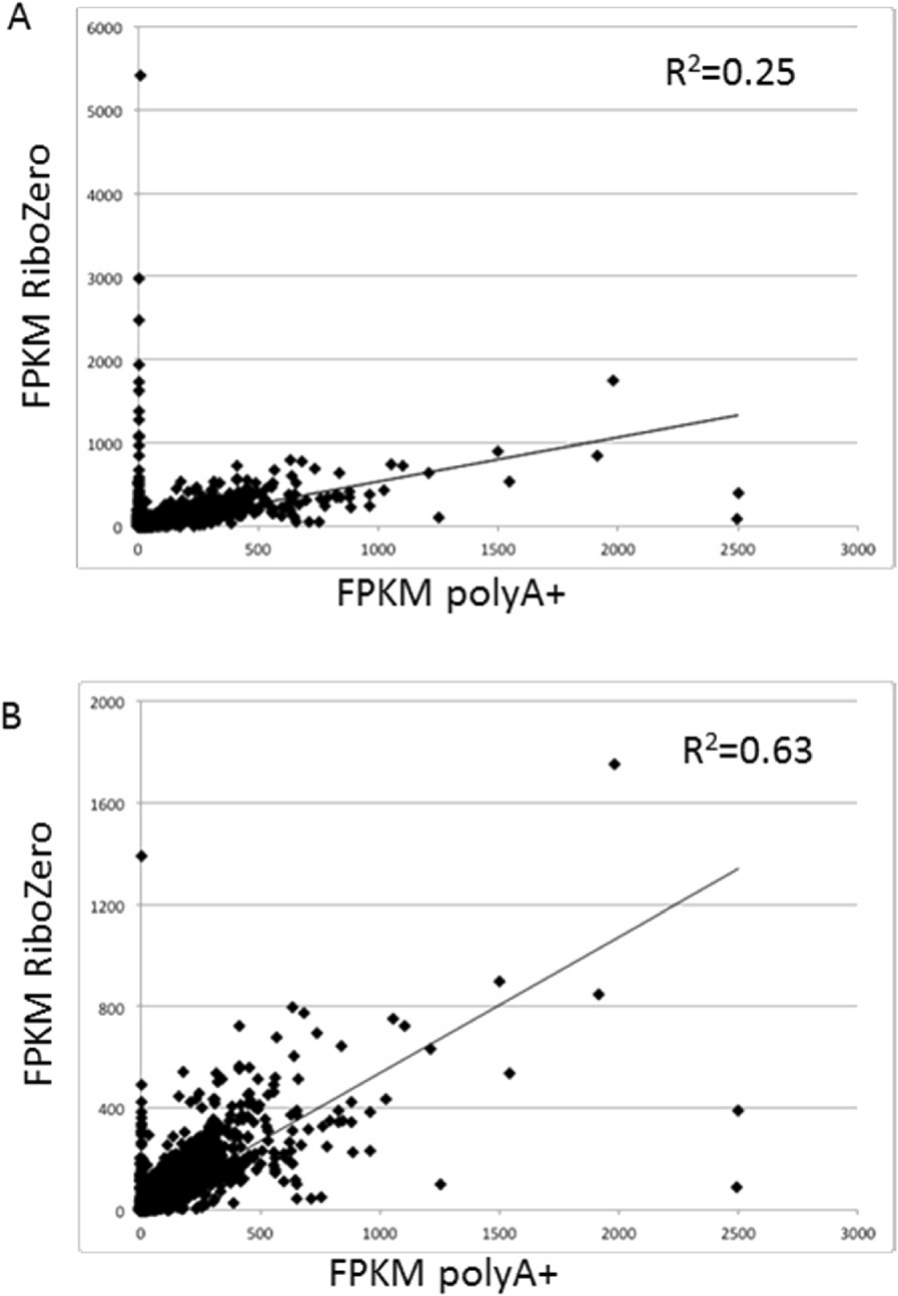
**Plot of expression values obtained in RiboMinus versus polyA+ selected RNA in the F118 control sample. A)** Plot of expression values of all samples in both the RNA selection methods. **B)** Plot of expression values where all samples in both the selection methods likely to encode organellar or ribosomal sequences were removed through automated and manual curation of aberrant ratios between the two RNA selection methods.

Ribosomal RNA-depleted samples obtained from plants infected with pathogenic bacteria should provide simultaneous information on gene expression from both the plant and the pathogen. Thus, one of the goals of this work was to compare two methods for rRNA removal before transcriptome analysis using RNAseq: ribosomal RNA depletion with Ribozero® (Epicentre) and PolyA+ selection to enrich for mRNA. Starting from the same total RNA samples used above for polyA+ selection and RNA-seq, we performed ribosomal RNA depletion with Ribozero®, after which Illumina RNA-seq libraries were constructed and sequenced. With respect to *S. commersonii* transcripts, PolyA+ vs rRNA-depleted transcriptomes should only differ in organellar genes that remain in the latter procedure and the fact that we were using root samples -devoid of chloroplasts- should minimize this problem. Our first comparisons of transcript expression levels for each condition obtained with both methodologies showed distorted ratios for a number of genes that are most likely organellar in origin, thereby biasing the FPKM ratios of expression abundances obtained from PolyA+ vs rRNA-depleted libraries. Thus, we searched the predicted DM proteome and the *S. commersonii* lineage-specific transcripts using the annotated DM chloroplast and mitochondrial genome sequences [69] and removed genes with high sequence identity and coverage to the organellar genomes. Surprisingly, organellar-derived transcripts remained, which upon close examination, had not met our initial identity and coverage cutoff criteria using the annotated DM organellar genes. Therefore, we decreased the stringency for identity and coverage cutoffs and broadened our query sequences to include the *A. thaliana* organelles to further define potato and *S. commersonii* genes with organellar identity. Gene expression comparison (in FPKMs) between PolyA+ vs rRNA-depleted samples in a set of libraries is shown in Figure 7. Transcripts showing aberrant ratios between the two mRNA selection methods were curated manually and those likely to encode organellar or bacterial sequences were removed. While we were able to remove the majority of outliers, several genes/transcripts lacked congruency in FPKM values between the two isolation methods, which affected identification of differentially expressed genes. Some of these outliers were likely due to still imperfect filtering for organellar genes or to other non-PolyA+ RNAs. Thus, we used Spearman Rank Correlation to determine the concordance of expression values in the PolyA+ vs rRNA-depleted samples (Additional file 1: Table S6). Indeed, the high degree of concordance between the samples using the Spearman Rank Correlation shows that for the most part, these two methods are comparable (Figure 7). However, the outliers and variation in the lowly expressed genes negatively affect both the Pearson Correlation Coefficient (Additional file 1: Table S6) and identification of differentially expressed genes. These results suggest that the technical issues with organellar and bacterial mRNAs that are not removed using rRNA depletion will not provide identical results as through conventional polyA+ selection, in which contamination from bacterial and organellar mRNAs is minimized.

## Conclusions

Sequence of the transcriptomes of two accessions of *S. commersonii* and *S. commersonii* challenged with *R. solanacearum* are described for the first time, providing a valuable tool for plant breeding programs. Two percent of all *S. commersonii* genes were differentially expressed after pathogen infection. From these, hormone related genes indicated that both ET and JA were induced in the susceptible accession F97, but not in the resistant accession F118. In contrast, SA-related genes were down-regulated in both accessions after pathogen infection. We compared two different methods to remove ribosomal RNA from the plant samples: PolyA+ selection to enrich for mRNA vs ribosomal RNA depletion using Ribozero®, and determined that conventional PolyA+ selection of mRNA minimizes bacterial and organellar contamination when compared to rRNA depletion.

## Methods

### Plant material, bacterial strains and inoculation procedures

Two individuals from a segregating population of the wild potato *S. commersonii* that showed marked differences in resistance against *R. solanace*arum were used in this study (F97, susceptible and F118, resistant) [22]. Plants were propagated *in vitro* in Murashige and Skoog (MS) medium for two weeks, transferred to a soil mix and grown in a greenhouse for one week with a 12 hour light photoperiod and temperatures kept at 22 to 25°C. Plants were then transferred to a growth chamber at 27°C and 65% relative humidity (RH) for acclimation one week prior to inoculation with *R. solanacearum*.

The highly aggressive *R. solanacearum* strain UY031 (phylotype IIB, sequevar 1-2) isolated from potato in Uruguay [4,70], which had been genetically modified to carry the *lux*-operon under control of the *eps* promoter [35], was used. Prior to inoculation, potato roots from both water controls and pathogen-inoculated plants were injured with a 1 ml pipette tip. Inoculations were performed as described [19] by drenching the soil with a concentration of 10^7^ cfu/ml of *R. solanacearum* per gram of soil, and control plants were drenched with water. After inoculation, plants were kept in the inoculation chamber with the same conditions as described above.

### Tissue collection, RNA extraction and mRNA isolation

RNA was extracted from asymptomatic plants of *S. commersonii* three to four days after inoculation (dai). Root samples were collected by measuring one centimeter of root from the base of the stem. Their bacterial content was estimated by measuring luminescence with an FB12 luminometer (Berthold Detection Systems) normalizing by tissue weight. Samples with similar bacterial populations (estimated at approximately 10^5^ colony forming units per g of tissue) were pooled together and flash frozen in liquid nitrogen. Pools of root tissue containing at least seven plants per genotype per treatment (F97 and F118, inoculated and non-inoculated controls) were obtained from three independent biological trials (biological replicas). After tissue grinding in liquid nitrogen with a cold mortar and pestle, total root RNA was extracted from these biological replicas using the NucleSpin® RNA plant extraction kit from Macherey-Nagel following manufacturer’s instructions. Total RNA was treated with DNase I. Quality and abundance of RNA was verified using a Bioanalyzer 2100 (Agilent Technologies) and only samples with an RNA Integrity Number (RIN) over 8 were used.

For ribosomal RNA depletion, samples were treated with rRNA removal solution from the Ribo-Zero(™) magnetic kit for plant seed/root (Epicentre) following manufacturer’s instructions. Briefly, 2.5 μg of total RNA from each sample was treated with both the rRNA removal solution from the plant seed/root and gram-negative bacteria kits, in a 50:50 ratio. mRNA-enriched fractions from the biological replicas were separately subjected to deep sequencing on an Illumina-Solexa Genome Analyzer II using multiplexing and the kits specially adapted to obtain 76-nucleotide paired-end reads. For samples where PolyA was isolated, the procedure was performed directly following the Truseq Illumina RNAseq procedure.

Reads have been deposited in the National Center for Biotechnology Information Sequence Read Archive under BioProject ### (to be made available upon publication).

### *S. commersonii* transcriptome assembly, functional annotation and expression abundances

FASTQC (v 0.10.1) was used to determine the quality of the RNA-seq data (http://www.bioinformatics.babraham.ac.uk/projects/fastqc/). Reads were trimmed and cleaned of Illumina adaptors and low quality sequences using the FASTX tool kit (v 0.0.13) [71] and Cutadapt (v 1.2.1) [72]. Sequences with a quality score equal or greater than 20 and a minimum length of 50 nt were retained. Cleaned reads were mapped to the *S. tuberosum* Group Phureja DM genome [36] using Tophat (SAMTools v 0.1.12/Bowtie v 0.12.7/Tophat v 1.4.1) [37,73,74] and unmapped reads were retained. A second round of cleaning was performed using FASTX tool kit (v 0.0.13) [71]. Cleaned unmapped sequences were assembled using the Oases assembler (Velvet v 1.2.08/ Oases v 0.2.08) with a k-mer length of 31 [38,75]. Oases-derived contigs were filtered out for low complexity sequences as described previously [41]. The transcript assembly was screened for non-*S. commersonii* sequences using three filters. First, assembled transcripts were aligned using BLASTX (WUBLAST v 2.2.6) against UniRef100 [39,40] and transcripts with a match at ≥ 70% identity and over ≥ 85% coverage to bacterial, fungal, viral, viroid, arthropod, stramenopile or human sequences were removed as contaminants (626). Manual review revealed additional contaminants may be present in the *S. commersonii* transcript assemblies and as a second filter, the transcripts with an E-value match of < 1e-10 to any non-Viridiplantae UniRef100 entry was removed as a potential contaminant (1,144). As a third filter, any remaining *S. commersonii* transcripts lacking of UniRef100 annotation were searched against the DM predicted proteome [36] and transcripts with *<* 50% identity, *<* 50% coverage with an E-value > 1e-05 to a predicted potato protein were removed (5,644).

Gmap (v 20130331) was used to align assembled transcripts to the *S. tuberosum* Group Phureja DM genome [36,42]. Un-aligned assemblies were retained and these assemblies were used to build a *S. commersonii* reference sequence. These novel transcripts, along with their annotation, are available as Additional file 2. Transcripts sequences were concatenated using a unigene set defined as all of the single isoform transcripts and the representative transcript (the longest isoform) for those assemblies in which more than one isoform were generated by Oases [38,41]. The concatenated transcript sequences (CTS) were added to the *S. tuberosum* Group Phureja DM genome as an extra chromosome (*S. commersonii* lineage specific). The DM genome and *S. commersonii* lineage-specific CTS were then used as a reference to map *S. commersonii* RNA-seq reads and calculate the expression abundances using Tophat (SAMTools v 0.1.12/Bowtie v 0.12.7/Tophat v 1.4.1) and Cufflinks (v 1.3.0), respectively; Cuffdiff was used to carry out differential expression analysis [37,43,73,74]. Genes were considered to be differentially expressed based on a FDR <0.05 and a four-fold change of transcript read abundance. We used these strict thresholds in order to select for relevant and robust differentially expressed genes.

For comparison of our transcriptomic data with previously defined potato transcriptional responses to stress and hormones [45], we selected genes annotated in the DM reference genome which were differentially expressed (FDR <0.05 and fold change >4) in non-inoculated vs *R. solanacearum*-challenged samples. We compared these genes to the biotic, abiotic and hormone potato response genes defined in the literature using a different threshold (FRKM > 1 and fold change >2) [45] and calculated the percentage of overlap of the different datasets.

For functional annotation, assembled transcripts were aligned using BLASTX (WUBLAST v 2.2.6) against UniRef100 [39,40] and an E-value cutoff of 1e-10 was used to transitively assign functional annotation. Predicted translations were generated using ESTscan (v 3.0.3) and GO associations were made by InterProScan (v 5.0.rc7) using an E-value cutoff of 1e-10 to assign similarity [76-78]. GO terms were further reduced to GO Slims terms using custom perl scripts.

## Additional files

Additional file 1: Table S1. Genes most highly expressed in S. commersonii roots. **Table S2.** Differentially-expressed genes in non-inoculated vs pathogen-inoculated roots of the resistant S. commersonii accession F118. **Table S3.** Differentially-expressed genes in non-inoculated vs pathogen-inoculated roots of the susceptible *S. commersonii* accession F97. **Table S4.** Differentially-expressed genes in non-inoculated roots of the F97 vs F118 accessions of S. commersonii. **Table S5.** Differentially-expressed genes in inoculated roots of the F97 vs F118 accessions of *S. commersonii.* **Table S6.** Spearman Rank Correlation to determine the concordance of expression values in polyA vs rRNA-depleted samples. Additional file 2:
**Dataset.**

## References

[1] Peeters N, Guidot A, Vailleau F, Valls M (2013). *Ralstonia solanacearum*, a widespread bacterial plant pathogen in the post-genomic era. Mol Plant Pathol.

[2] Coll NS, Valls M (2013). Current knowledge on the *Ralstonia solanacearum* type III secretion system. Microb Biotech.

[3] Priou S (2004). Integrated management of bacterial wilt and soil-borne diseases of potato in farmer communities of the inter-Andean valleys of Peru and Bolivia. Final Technical Report DFID-funded project CRF 7862(C).

[4] Genin S, Denny TP (2012). Pathogenomics of the *Ralstonia solanacearum* species complex. Ann Rev Phyto.

[5] Hong J, Ji P, Momol MT, Jones JB, Olson SM, Pradhanang P (2005). *Ralstonia solanacearum* detection in tomato irrigation ponds and weeds. Proceedings of the First International Symposium on Tomato Diseases: 2005.

[6] Qian YL, Wang XS, Wang DZ, Zhang LN, Zu CL, Gao ZL (2012). The detection of QTLs controlling bacterial wilt resistance in tobacco (*N. tabacum* L.). Euphytica.

[7] Carmeille ACC, Dintinger J, Prior P, Luisetti J, Besse P (2006). Identification of QTLs for *Ralstonia solanacearum* race 3-phylotype II resistance in tomato. Theor Appl Genet.

[8] Wang JF, Ho FI, Truong HTH, Huang SM, Balatero CH, Dittapongpitch V (2013). Identification of major QTLs associated with stable resistance of tomato cultivar ‘Hawaii 7996’ to *Ralstonia solanacearum*. Euphytica.

[9] Lebeau AGM, Daunay MC, Wicker E, Chiroleu F, Prior P, Frary A (2013). Genetic mapping of a major dominant gene for resistance to *Ralstonia solanacearum* in eggplant. Theor Appl Genet.

[10] Ben C, Debell F, Berges H, Bellec A, Jardinaud MF, Anson P (2013). MtQRRS1, an R-locus required for *Medicago truncatula* quantitative resistance to *Ralstonia solanacearum*. New Phytol.

[11] Deslandes L, Olivier J, Peeters N, Feng DX, Khounlotham M, Boucher C (2003). Physical interaction between RRS1-R, a protein conferring resistance to bacterial wilt, and PopP2, a type III effector targeted to the plant nucleus. PNAS USA.

[12] Deslandes L, Olivier J, Theulieres F, Hirsch J, Feng DX, Bittner-Eddy P (2002). Resistance to *Ralstonia solanacearum* in Arabidopsis thaliana is conferred by the recessive RRS1-R gene, a member of a novel family of resistance genes. PNAS USA.

[13] Deslandes L, Pileur F, Liaubet L, Camut S, Can C, Williams K (1998). Genetic characterization of RRS1, a recessive locus in *Arabidopsis thaliana* that confers resistance to the bacterial soilborne pathogen *Ralstonia solanacearum*. MPMI.

[14] Carputo D, Aversano R, Barone A, Di Matteo A, Iorizzo M, Sigillo L (2009). Resistance to *Ralstonia solanacearum* of Sexual Hybrids Between *Solanum commersonii* and *S. tuberosum*. American J Pot Res.

[15] Narancio R, Zorrilla P, Robello C, Gonzalez M, Vilaro F, Pritsch C (2013). Insights on gene expression response of a characterized resistance genotype of Solanum commersonii Dun. against *Ralstonia solanacearum*. European J Plant Pathol.

[16] Sequeira L, Rowe PR (1969). Selection and utilization of *Solanum phureja* clones with high resistance to different strains of *Pseudomonas solanacearum*. American J Potato Res.

[17] French ER, De Lindo L (1982). Resistance to *Pseudomonas solanacearum* in potato. Specificity and temperature sensitivity. Phytopath.

[18] Tung PX, Rasco ET, Vander Zaag P, Schmiediche P (1990). Resistance to *Pseudomonas solanacearum* in the potato: II. Aspects of host-pathogen-environment interaction. Euphytica.

[19] Zuluaga P, Ferreira V, Pianzzola MJ, Siri MI, Coll NS, Valls M (2014). A novel, sensitive method to evaluate potato germplasm for bacterial wilt resistance using a luminescent *Ralstonia solanacearum* reporter strain. MPMI.

[20] Hawkes JG, Bradshaw JE, Mackay GR (1994). Origins of cultivated potatoes and species relationships. Potato Genetics.

[21] Galván G, Fraguas F, Quirici L, Santos C, Silvera E, Siri M, Villanueva P, Raudiviniche L, González M, Torres D, Castillo A, Dalla Rizza M, Vilaró F, Gepp V, Ferreira F, Pianzzola MJ (2006). *Solanum Commersonii*: una especie con gran potencial para el mejoramiento genético de papa por resistencia a *Ralstonia Solanacearum*. Avances de investigación en recursos genéticos en el cono sur I.

[22] Gonzalez M, Galvan G, Siri MI, Borges A, Vilaro F (2013). Resistencia a la marchitez bacteriana de la papa en *Solanum commersonii* Dun. Agrociencia.

[23] Kim-Lee H, Moon JS, Hong YJ, Kim MS, Cho HM (2005). Bacterial wilt resistance in the progenies of the fusion hybrids between haploid of potato and *Solanum commersonii*. American J Potato Res.

[24] Laferriere LT, Helgeson JP, Allen C (1999). Fertile *Solanum tuberosum*+*S. commersonii* somatic hybrids as sources of resistance to bacterial wilt caused by *Ralstonia solanacearum*. Theor App Genet.

[25] Siri MI, Galván GA, Quirici L, Silvera E, Villanueva P, Ferreira F (2009). Molecular marker diversity and bacterial wilt resistance in wild *Solanum commersonii* accessions from Uruguay. Euphytica.

[26] Tao Y, Xie Z, Chen W, Glazebrook J, Chang HS, Han B (2003). Quantitative nature of *Arabidopsis* responses during compatible and incompatible interactions with the bacterial pathogen *Pseudomonas syringae*. Plant Cell.

[27] Ren H, Gu G, Long J, Yin Q, Wu T, Song T (2006). Combinative effects of a bacterial type-III effector and a biocontrol bacterium on rice growth and disease resistance. J Biosci.

[28] Gao L, Tu Jin Z, Millett BP, Bradeen JM (2013). Insights into organ-specific pathogen defense responses in plants: RNA-seq analysis of potato tuber-Phytophthora infestans interactions. BMC Genomics.

[29] Baebler S, Krecic-Stres H, Rotter A, Kogovsek P, Cankar K, Kok EJ (2009). PVY(NTN) elicits a diverse gene expression response in different potato genotypes in the first 12 h after inoculation. Mol Plant Pathol.

[30] Draffehn AM, Li L, Krezdorn N, Ding J, Lübeck J, Strahwald J, Muktar MS, Walkemeier B, Rotter B, Gebhardt C (2013). Comparative transcript profiling by SuperSAGE identifies novel candidate genes for controlling potato quantitative resistance to late blight not compromised by late maturity. Frontiers Plant Sci.

[31] Hu J, Barlet X, Deslandes L, Hirsch J, Feng DX, Somssich I (2008). Transcriptional responses of *Arabidopsis thaliana* during wilt disease caused by the soil-borne phytopathogenic bacterium. Ralstonia solanacearum. PLoS One.

[32] Ishihara T, Mitsuhara, I, Takahashi H, Nakaho K. Transcriptome analysis of quantitative resistance-specific response upon *Ralstonia solanacearum* infection in tomato. PlosOne 2012; doi:10.1371/journal.pone.0046763. 10.1371/journal.pone.0046763 PMC3465262 23071630

[33] Esposito N, Ovchinnikova OG, Barone A, Zoina A, Holst O, Evidente A (2008). Host and non-host plant response to bacterial wilt in potato: role of the lipopolysaccharide isolated from *Ralstonia solanacearum* and molecular analysis of plant-pathogen interaction. Chem Biodivers.

[34] Mortazavi A, Williams BA, McCue K, Schaeffer L, Wold B (2008). Mapping and quantifying mammalian transcriptomes by RNA-Seq. Nat Methods.

[35] Monteiro F, Genin S, van Dijk I, Valls M (2012). A luminescent reporter evidences active expression of *Ralstonia solanacearum* type III secretion system genes throughout plant infection. Microbiology.

[36] Consortium TPGS (2011). Genome sequence and analysis of the tuber crop potato. Nature.

[37] Trapnell C, Pachter L, Salzberg SL (2009). TopHat: discovering splice junctions with RNA-Seq. Bioinformatics.

[38] Schulz MH, Zerbino DR, Vingron M, Birney E (2012). Oases: robust de novo RNA-seq assembly across the dynamic range of expression levels. Bioinformatics.

[39] Suzek BE, Huang H, McGarvey P, Mazumder R, Wu CH (2007). UniRef: comprehensive and non-redundant UniProt reference clusters. Bioinformatics.

[40] Altschul SF, Gish W, Miller W, Myers EW, Lipman DJ (1990). Basic local alignment search tool. J Mol Biol.

[41] Góngora-Castillo E, Fedewa G, Yeo Y, Chappell J, DellaPenna D, Buell CR (2012). Genomic approaches for interrogating the biochemistry of medicinal plant species. Methods Enzymol.

[42] Wu TD, Watanabe CK (2005). GMAP: a genomic mapping and alignment program for mRNA and EST sequences. Bioinformatics.

[43] Trapnell C, Williams BA, Pertea G, Mortazavi A, Kwan G, van Baren MJ (2010). Transcript assembly and quantification by RNA-Seq reveals unannotated transcripts and isoform switching during cell differentiation. Nat Biotech.

[44] Chandran D, Rickert J, Huang Y, Steinwand MA, Marr SK, Wildermuth MC (2014). Atypical E2F transcriptional repressor DEL1 acts at the intersection of plant growth and immunity by controlling the hormone salicylic acid. Cell Host Microbe.

[45] Massa AN, Childs KL, Buell CR (2013). Abiotic and Biotic Stress Responses in *Solanum tuberosum* Group Phureja DM1-3 516 R44 as Measured through Whole Transcriptome Sequencing. Plant Gen.

[46] Beffa RS, Neuhaus JM, Meins F (1993). Physiological compensation in antisense transformants: specific induction of an “ersatz” glucan endo-1,3-beta-glucosidase in plants infected with necrotizing viruses. PNAS USA.

[47] Kombrink E, Schroder M, Hahlbrock K (1988). Several “pathogenesis-related” proteins in potato are 1,3-beta-glucanases and chitinases. PNAS USA.

[48] Melchers LS, Apotheker-de Groot M, van derKnaap JA, Ponstein AS, Sela-Buurlage MB, Bol JF (1994). A new class of tobacco chitinases homologous to bacterial exo-chitinases displays antifungal activity. Plant J: for cell and molecular biology.

[49] Jordá L, Coego A, Conejero V, Vera P (1999). A Genomic Cluster Containing Four Differentially Regulated Subtilisin-like Processing Protease Genes Is in Tomato Plants. J Biol Chem.

[50] Jorda L, Conejero V, Vera P (2000). Characterization of P69E and P69F, two differentially regulated genes encoding new members of the subtilisin-like proteinase family from tomato plants. Plant Physiol.

[51] Villanueva J, Canals F, Prat S, Ludevid D, Querol E, Aviles FX (1998). Characterization of the wound-induced metallocarboxypeptidase inhibitor from potato. cDNA sequence, induction of gene expression, subcellular immunolocalization and potential roles of the C-terminal propeptide. FEBS Lett.

[52] Gonzalez-Lamothe R, El Oirdi M, Brisson N, Bouarab K (2012). The conjugated auxin indole-3-acetic acid-aspartic acid promotes plant disease development. Plant Cell.

[53] Staswick PE, Serban B, Rowe M, Tiryaki I, Maldonado MT, Maldonado MC (2005). Characterization of an *Arabidopsis* Enzyme Family That Conjugates Amino Acids to Indole-3-Acetic Acid. Plant Cell Online.

[54] Amano Y, Tsubouchi H, Shinohara H, Ogawa M, Matsubayashi Y (2007). Tyrosine-sulfated glycopeptide involved in cellular proliferation and expansion in *Arabidopsis*. PNAS USA.

[55] Gutierrez L, Mongelard G, Floková K, Păcurar DI, Novák O, Staswick P (2012). Auxin Controls *Arabidopsis* Adventitious Root Initiation by Regulating Jasmonic Acid Homeostasis. Plant Cell Online.

[56] Igarashi D, Tsuda K, Katagiri F (2012). The peptide growth factor, phytosulfokine, attenuates pattern-triggered immunity. Plant J: for cell and, molecular biology.

[57] Mazarei M, Lennon KA, Puthoff DP, Rodermel SR, Baum TJ (2003). Expression of an *Arabidopsis* phosphoglycerate mutase homologue is localized to apical meristems, regulated by hormones, and induced by sedentary plant-parasitic nematodes. Plant Mol Biol.

[58] Van Ooijen G, Lukasik E, Van Den Burg HA, Vossen JH, Cornelissen BJ, Takken FL (2010). The small heat shock protein 20 RSI2 interacts with and is required for stability and function of tomato resistance protein I-2. Plant J: for cell and, molecular biology.

[59] Maimbo M, Ohnishi K, Hikichi Y, Yoshioka H, Kiba A (2007). Induction of a small heat shock protein and its functional roles in *Nicotiana* plants in the defense response against *Ralstonia solanacearum*. Plant Physiol.

[60] Jiang HW, Liu MJ, Chen IC, Huang CH, Chao LY, Hsieh HL (2010). A glutathione S-transferase regulated by light and hormones participates in the modulation of *Arabidopsis* seedling development. Plant Physiol.

[61] Dean JD, Goodwin PH, Hsiang T (2005). Induction of glutathione S-transferase genes of *Nicotiana benthamiana* following infection by *Colletotrichum destructivum* and C. orbiculare and involvement of one in resistance. J exp botany.

[62] Mur LA, Aubry S, Mondhe M, Kingston-Smith A, Gallagher J, Timms-Taravella E (2010). Accumulation of chlorophyll catabolites photosensitizes the hypersensitive response elicited by *Pseudomonas syringae* in *Arabidopsis*. New Phytol.

[63] Meng X, Zhang S (2013). MAPK cascades in plant disease resistance signaling. Ann Rev Phytopatho.

[64] Carter CJ, Thornburg RW (2004). Tobacco nectarin V is a flavin-containing berberine bridge enzyme-like protein with glucose oxidase activity. Plant Physiol.

[65] Schilmiller AL, Koo AJ, Howe GA (2007). Functional diversification of acyl-coenzyme A oxidases in jasmonic acid biosynthesis and action. Plant Physiol.

[66] X-y Z, Spivey NW, Zeng W, Liu P-P, Fu ZQ, Klessig DF (2012). Coronatine promotes *Pseudomonas syringae* virulence in plants by activating a signaling cascade that inhibits salicylic acid accumulation. Cell Host Microbe.

[67] Hirsch J, Deslandes L, Feng DX, Balague C, Marco Y (2002). Delayed Symptom Development in ein2-1, an Arabidopsis Ethylene-Insensitive Mutant, in Response to Bacterial Wilt Caused by *Ralstonia solanacearum*. Phytopathology.

[68] D’Ovidio R, Mattei B, Roberti S, Bellincampi D (2004). Polygalacturonases, polygalacturonase-inhibiting proteins and pectic oligomers in plant-pathogen interactions. Bioch et biophys acta.

[69] Zhu W, Ouyang S, Iovene M, O’Brien K, Vuong H, Jiang J (2008). Analysis of 90 Mb of the potato genome reveals conservation of gene structures and order with tomato but divergence in repetitive sequence composition. BMC Genomics.

[70] Siri MI, Pianzzola MJ (2011). Genetic diversity and agressiveness of *Ralstonia solanacearum* strains causing bacterial wilt of potato in Uruguay. Plant Dis.

[71] Blankenberg D, Gordon A, Von Kuster G, Coraor N, Taylor J, Nekrutenko A (2010). Manipulation of FASTQ data with Galaxy. Bioinformatics.

[72] Martin M (2011). Cutadapt removes adapter sequences from high-throughput sequencing reads. EMBnet J.

[73] Langmead B, Trapnell C, Pop M, Salzberg S (2009). Ultrafast and memory-efficient alignment of short DNA sequences to the human genome. Genome Biol.

[74] Li H, Handsaker B, Wysoker A, Fennell T, Ruan J, Homer N (2009). The Sequence Alignment/Map format and SAMtools. Bioinformatics.

[75] Zerbino DR, Birney E (2008). Velvet: Algorithms for de novo short read assembly using de Bruijn graphs. Genome Res.

[76] Harris MA, Clark J, Ireland A, Lomax J, Ashburner M, Foulger R (2004). The Gene Ontology (GO) database and informatics resource. Nuc acids res.

[77] Iseli C, Jongeneel CV, Bucher P. ESTScan: a program for detecting, evaluating, and reconstructing potential coding regions in EST sequences. Proceedings/International Conference on Intelligent Systems for Molecular Biology1999;138-148. 10786296

[78] Quevillon E, Silventoinen V, Pillai S, Harte N, Mulder N, Apweiler R (2005). InterProScan: protein domains identifier. Nucl acids res.

